# Drug-Induced Thrombocytopenia Due to Nintedanib during Treatment of Idiopathic Pulmonary Fibrosis

**DOI:** 10.3390/medicina59050999

**Published:** 2023-05-22

**Authors:** Igor Dumic, Antonios Charokopos, Angadabir Parmar, Christopher R. Grant, Ronin Joshua S. Cosiquien, Marilia Dagnon da Silva, Emilia Petcu

**Affiliations:** 1 Mayo Clinic College of Medicine and Science, Rochester, MN 55905, USA; charokopos.antonios@mayo.edu (A.C.);; 2 Department of Hospital Medicine, Mayo Clinic Health System, Eau Claire, WI 54703, USA; 3 Department of Pulmonary Medicine and Critical Care, Mayo Clinic Health System, Eau Claire, WI 54703, USA; 4 Department of Internal Medicine, University of California, Irvine, CA 92697, USA; 5 University of Minnesota, Minneapolis, MN 55455, USA; cosiq001@umn.edu; 6 Municipal University of São Caetano do Sul—USCS Bela Vista, São Paulo 09521-160, Brazil

**Keywords:** drug-induced thrombocytopenia, idiopathic pulmonary fibrosis, Nintedanib

## Abstract

Nintedanib is a tyrosine kinase inhibitor that was approved for the treatment of patients with idiopathic pulmonary fibrosis in 2014. The most common side effect of Nintedanib is diarrhea, and thrombocytopenia is a rare side effect of Nintedanib. The exact mechanism is unknown, and the literature lacks case reports of this phenomenon. Here, we report the case of a patient who developed thrombocytopenia 12 weeks after starting treatment with Nintedanib. The patient underwent an extensive work up for infectious, hematological, autoimmune, and neoplastic diseases. The patient’s thrombocytopenia resolved following cessation of Nintedanib. This case is significant as it reports a rare side effect that might have detrimental consequences if not recognized and treated timely. Additionally, the onset of thrombocytopenia was delayed, 3 months after the initiation of Nintedanib. We also highlight the various literature regarding drug-induced thrombocytopenia and explore the necessary work-up needed to exclude other potential diagnoses. We hope to advocate for multidisciplinary teams to be aware of patients with pulmonary fibrosis on Nintedanib so that this adverse effect can be recognized promptly.

## 1. Introduction

Idiopathic pulmonary fibrosis (IPF) is a chronic progressive lung disease manifested by progressive fibrosis of the lung parenchyma, not caused by acute infection or cancer, which eventually leads to the loss of lung compliance resulting in decreased lung volume, respiratory failure, and death [1,2]. The incidence of IPF is 2.8–9.3 per 100,000 per year in North America and Europe with significantly lower rates in Asia and South America [1]. Patients can present with vague symptoms including progressive dyspnea, non-productive cough, and/or hypoxia [1]. Traditionally, IPF is diagnosed in a methodical manner, with a thorough laboratory evaluation to evaluate autoimmune etiologies. Subsequently, radiographic findings will demonstrate basilar-predominance, traction bronchiectasis, and honeycombing features on CT imaging. Pulmonary function tests (PFTs) will also demonstrate a restrictive pattern. The mortality from IPF is high, and has a 5-year transplant-free survival rate of 45% [3,4] with very limited treatment options, which include pirfenidone, an antifibrotic, and Nintedanib.

Nintedanib is a tyrosine kinase inhibitor. Its mechanism of action includes blockade of vascular endothelial growth factor (VEGF) pathway, platelet derived growth factor (PDGF), and fibroblast growth factor (FGF) receptors [4,5]. While it was initially developed as an antineoplastic agent, it was discovered that it also exhibits anti-fibrotic properties by reducing fibroblast activity [4,6].

TOMORROW (To Improve Pulmonary Fibrosis with BIBF 1120) trial was a phase II study that demonstrated the promising benefit of Nintedanib in patients with IPF and was effective in reducing FVC decline. This was followed by two phase III studies (IMPULSIS 1 and 2) that were multi-centered randomized controlled trials that confirmed that Nintedanib at the dose of 150 mg orally two times per day led to a 5–10% reduce in decline in forced vital capacity (FVC) in the treatment group compared to placebo [7]. This led to the United States Food and Drug Administration (FDA) approval of Nintedanib in 2020 for the treatment of IPF.

Thrombocytopenia as a side effect of Nintedanib has rarely been reported [5,8,9]. Drug-induced thrombocytopenia is an important side effect of Nintedanib that clinicians should be aware of. Here, we report the case of a patient who developed thrombocytopenia 12 weeks after starting treatment with Nintedanib. We hope to raise awareness amongst multidisciplinary teams including pulmonologists, hematologists, internists, and primary care providers so they can be aware of this rare and potentially serious side effect that can be fatal if left unrecognized.

## 2. Case Presentation

**Figure 1:**
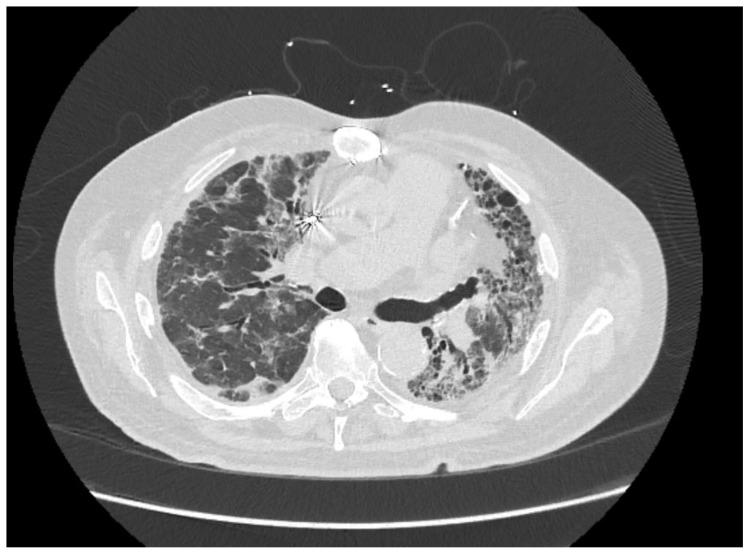
Axial view of chest computed tomography (CT) at the subcarinal level, showing advanced left lung fibrosis (with traction bronchiectasis and reticulation) and more acute right lung inflammation (ground glass opacities mixed with traction bronchiectasis).

**Figure 2:**
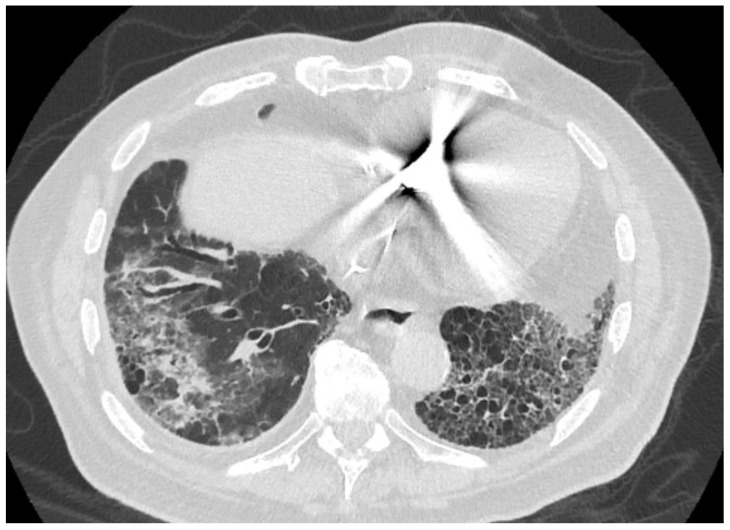
Axial view of chest computed tomography (CT) demonstrates typical honeycombing in the left lower lobe and traction bronchiectasis in the right lower lobe. The groundglass and consolidative opacity seen in the right lower lobe—which is not common in UIP/IPF—has been attributed to possible mild focal alveolar hemorrhage in the setting of thrombocytopenia or microaspiration inflammatory changes.

A 78-year-old man, who was diagnosed with IPF two years prior, was admitted for evaluation of low platelet count of 61.000 μL associated with easy bruising. The patient had a history of coronary artery disease and ischemic cardiomyopathy for which he was taking aspirin and carvedilol, atrial fibrillation that was rate-controlled with carvedilol, and he was not anticoagulated due to prior gastrointestinal bleeding. IPF was diagnosed two years prior based on symptoms of worsening shortness of breath, exertional hypoxia, and radiologic findings on chest computed tomography (CT) (Figure 1 and Figure 2).

**Figure 3:**
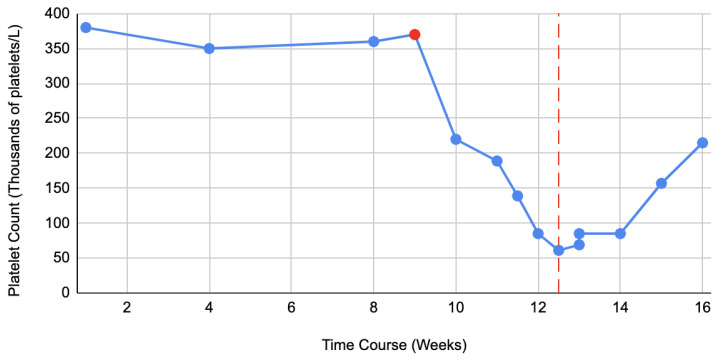
Demonstrates trend in platelet counts from the time of initiation of Nintedanib until it is stopped (red line). The red dot points towards the time when dose of Nintedanib was decreased from 150 mg orally two times per day to 150 mg orally daily due to gastrointestinal side effects.

The patient lived in rural Wisconsin, did not smoke, use illicit drugs, or drink alcohol. He did not take any other over-the-counter medications or herbal supplements. He did not have any family history of lung disease and denied any occupational exposure to toxic fumes. For IPF, he was treated with mycophenolate mofetil for a year; however, due to disease progression, he transitioned to Nintedanib 150 mg orally two times a day. Eight weeks into the treatment he started having nausea, diarrhea, and weight loss which was attributed to the Nintedanib and dose was reduced to 150 mg daily (Figure 3). With this change his gastrointestinal symptoms resolved, and he continued to take the medication for the next four weeks. Approximately 12 weeks after initiation of Nintedanib, the patient noted more bruises on his upper and lower extremities. They would appear spontaneously without trauma. He denied any hemoptysis, hematemesis, hematochezia, melena, or hematuria. He visited the emergency department where he was found to have a platelet count of 88.000/μL and was admitted for further investigation.

Laboratory work up on admission demonstrated normal hemoglobin and white blood cell count. Pseudothrombocytopenia was excluded and peripheral blood smear showed isolated decrease in platelet count with normal appearing neutrophils and erythrocytes. Coagulation studies, including prothrombin time, activated partial thromboplastin time, D dimer, and fibrinogen were normal as well. Lactate dehydrogenase (LDH), C reactive protein (CRP), and Erythrocyte sedimentation rate (ESR) were all within normal range. Electrolytes, liver function tests, and renal function were normal, as well as thyroid stimulating hormone and cortisol levels. Infectious work up was negative for Hepatitis B (HBV) and Hepatitis C (HCV), Epstein–Barr virus (EBV), Cytomegalovirus (CMV), and Human Immunodeficiency Virus (HIV). Since the patient lived in tick-borne disease endemic area of the United States, further infectious work up for Lyme disease, *Anaplasma phagocytophilum*, *Babesia microti*, and *Ehrlichia chaffeensis* was performed and was negative. Protein electrophoresis was negative for any monoclonal spikes. *Helicobacter pylori* stool antigen test was negative as well as anti-nuclear antibodies (ANA).

Since broad work up for infectious, autoimmune, hematologic and malignant conditions were negative, it was suspected that the thrombocytopenia might be drug related. A careful review of patient’s medications was conducted. Nintedanib was the most recently added, and the patient had tolerated aspirin and carvedilol for many years prior without any side effects. Hence, Nintedanib was discontinued, and patient’s platelet count gradually resolved as illustrated in Figure 3. Bruising subsided and eventually resolved, and the patient continued to take aspirin and carvedilol without any side effects.

## 3. Discussion

Thrombocytopenia is defined as a platelet count below the lower limit of normal (i.e., <150,000/μL for adults). It can be further divided into mild (platelet count 100,000 to 150,000/μL), moderate (50,000 to 99,000/μL), and severe (<50,000/μL) [10]. However, it is not only an absolute platelet number, but also the trend in platelets values that should be taken into consideration when evaluating the risk of bleeding in a patient as well as other factors that might contribute to overall bleeding risk. For example, for the same platelet number, patients with immune thrombocytopenia might have lower bleeding risk than those with an acute decrease in platelets such as patients who are receiving chemotherapy or have thrombocytopenia secondary to aplastic anemia. If the patient is on antiplatelet therapy or anticoagulation, it further increases the risk of bleeding. Severe thrombocytopenia has the highest risk of bleeding, and implies a greater likelihood for needing treatment, but correlation between platelet count and increased risk of bleeding depends on underlying condition and patient specifics risks [11]. Piel-Julian et al. demonstrated that platelet count, female sex, and exposure to non-steroidal anti-inflammatory drugs (NSAID) are risk factors for major bleeding [12].

Approach to thrombocytopenia should begin with the exclusion of pseudothrombocytopenia. Pseudothrombocytopenia is an in vitro phenomenon that occurs due to platelet clumping secondary to incompletely mixed, inadequately anticoagulated, or specimens that were exposed to dipotassium ethylenediaminetetraacetic acid (EDTA), resulting in laboratory error [13]. It is prevented by drawing blood in a tube that does not contain dipotassium EDTA. Once this is ruled out, congenital (such as Alport syndrome and Fanconi anemia) and acquired disorders are considered. Our patient was an elderly man and congenital causes were ruled out based on his age and prior normal platelet count throughout his life.

Acquired thrombocytopenias have a wide differential diagnosis including autoimmune diseases, drugs, infectious agents, nutritional deficiencies, neoplasia, disseminated intravascular coagulation, pregnancy-related, splenomegaly-related, and microangiopathies [14]. In adult populations, immune thrombocytopenia is the most common cause of thrombocytopenia [14] followed by drug-induced thrombocytopenia (DIT). DIT is common but is a diagnosis of exclusion, and for timely diagnosis the clinicians must have high index of suspicion and obtain detailed medication history to identify the inciting drug [15,16,17,18].

Our patient resided in Wisconsin, which is an endemic region for tick borne diseases. Hence, we were particularly careful to exclude these infections. Among these infections that can present with thrombocytopenia, particularly common are anaplasmosis [19] and babesiosis [20]. In anaplasmosis, thrombocytopenia is the most common laboratory abnormality, and in patients with *Babesia microti* infection, presence of thrombocytopenia below 50.000/μL is associated with splenic rupture [19,20]. Myelodysplastic neoplasm (MDS), previously known as myelodysplastic syndrome, is a well-known cause of thrombocytopenia but must be diagnosed with bone marrow biopsy which is an invasive procedure [21]. It usually affects the elderly, so our patient was in the age group that is frequently affected by MDS. We did not suspect MDS since thrombocytopenia was subacute in onset and other cell lines were not affected. However, if withdrawal of Nintedanib did not result in recovery of platelet count, a bone marrow biopsy would have been the next step.

In critically ill patients, disseminated intravascular coagulation is a concerning cause of thrombocytopenia, which has a particularly high mortality [22]. Individuals, such as our patient, with isolated thrombocytopenia are more likely to have immune thrombocytopenia or DIT whereas in acutely ill, hospitalized patients, thrombocytopenia is more likely to be due to platelet consumption, dilution, bone marrow suppression and/or sepsis/infection [16,23]. Finally, splenomegaly related thrombocytopenia encountered in cirrhosis, and microangiopathies causing thrombocytopenia (hemolytic uremic syndrome) are other entities that were considered but were ruled out in our patient [24,25]. More than 300 drugs have been identified as possible causes for drug-induced thrombocytopenia, including chemotherapy agents (oxaliplatin), antibiotics (such as linezolid, trimethoprim/sulfasalazine, vancomycin, quinidine), antiepileptics (carbamazepine), and antidepressants (mirtazapine). Heparin induced thrombocytopenia is a separate and unique cause of acquired thrombocytopenia [15,16,17,18], which is significantly easier to confirm given that heparin-induced thrombocytopenia is typically due to antibodies directed against platelet-factor 4, which is able to be detected due to the sensitivity with specific assays [17].

Nintedanib is a multi-kinase inhibitor with activity against FGFR, PDGFR, and VEGFR. Nintedanib binds competitively to the adenosine triphosphate (ATP), the binding pocket of these receptors, and blocks the intracellular signaling crucial for the proliferation, migration, and transformation of fibroblasts [4,5,8]. It is FDA-approved for idiopathic pulmonary fibrosis and systemic sclerosis-associated interstitial lung disease. The most common side effects noted in clinical trial and postmarketing reports were diarrhea in 62% of patients, nausea in 25%, vomiting in 12%, and weight loss in 10% [4,7,8]. Thrombocytopenia was a very rare side effect that was reported in clinical trials. Bleeding complications during Nintedanib therapy occurred in 10% of the patients but this was not related to the thrombocytopenia that was noted in less than 1% of patients [7,8]. Due to these findings the manufacturer advised particular attention for patients who are on anticoagulants and antiplatelet medication concomitantly to treatment with Nintedanib. Our patient was taking aspirin due to the presence of coronary artery disease and atrial fibrillation, and it might have contributed to easy bruising. In our patient, thrombocytopenia did not develop while he was taking the dose of 150 mg two times a day, but later when the dose was 150 mg daily which suggests that thrombocytopenia is a dose independent side effect. In our patient, it occurred about 3 months after initiation of the medication, which is different than in the case report by Y Ochi et al. who reported a patient with Nintedanib induced thrombocytopenia that developed one month after the treatment initiation and reached nadir of 14.000/μL after 8 months of therapy [9].

The association between Nintedanib and thrombocytopenia has not been well reported since this is a rare side effect and Nintedanib is a newly approved medication. We searched PubMed database using the key words: Nintedanib and thrombocytopenia. Our search yielded 10 articles of which none was related to thrombocytopenia. Further search using Google Scholar resulted in only one case that reported this rare side effect [9]. Other multi-target tyrosine kinase inhibitors, such as sunitinib and sorafenib have been shown to cause significant hematological adverse events effects, including thrombocytopenia [26,27]. It may be that Nintedanib-mediated inhibition of PDGFR could mechanistically explain the platelet suppression [28]. For these two agents, thrombocytopenia was demonstrated to be either due to bone marrow suppression or secondary to immune mechanisms.

There is new evidence that some drugs (aspirin, vancomycin, lovastatin, cisplatin, methotrexate, and doxorubicin) might cause thrombocytopenia via pro apoptotic signaling including Ca + 2 signaling, mitochondrial depolarization, and phosphatidylserine exposure in platelets [29]. A major limitation of the available evidence is that nearly all the studies were conducted in vitro using washed platelets, missing in vivo studies [29]. Additionally, not all patients treated with these drugs experience thrombocytopenia so further studies are needed to evaluate the pro apoptotic effect of some drugs on platelet count [29]. At this moment, there is no evidence to support that DIT due to Nintedanib is caused by this mechanism.

Drug-induced immune thrombocytopenia occurs within 1–2 weeks of daily exposure to a new drug and thrombocytopenia typically resolves within 5–7 days of drug discontinuation. Drug-induced thrombocytopenia with non-immune mechanisms such as bone marrow suppression can also occur, commonly affecting all blood cell lines [17,18]. In our patient, it occurred 3 months after the therapy was started, affected only platelets while other cell lines were unaffected, and resolved promptly within a week of medication cessation. The exact mechanism of thrombocytopenia remains unclear at this point.

With the advent of formulation of Nintedanib with antioxidants for biomedical applications, the authors are hopeful that we will find an improvement in oxidative stress and rare side effects such as immune thrombocytopenia. Numerous studies have demonstrated a benefit in antioxidant properties being beneficial. For instance, Mandegary et al. evaluated the hepatoprotective effect of silyamarin in individuals chronically exposed to hydrogen sulfide and found that there was significant effects of silymarin exposed chronically to hydrogen sulfide, denoting the modulatory effect of TNF-alpha leading to a reduction in AST, ALT, and ALP after 30 days of consumption [30] Studies have also reported a benefit in nanoparticles and green nanomaterials in delivering drugs to mitochondria and target cells, which represents a key benefit in assisting with IPF treatment [31]. Additional studies have led to the proposal of new nanoscale drug delivery systems to minimize the induced oxidative stress factors when formulating drugs such as Nintedanib [32]. IPF’s underlying mechanism stems from oxidative stress resulting in excessive tissue remodeling leading to further fibrosis [33]. Nintedanib itself has shown to exert beneficial effects on markers of oxidative stress independently [34]. We believe that there could be a synergistic benefit of combining antioxidants with Nintedanib to better create an optimal therapeutic effect in improving oxidative stress in IPF. Further equipoise exists on whether antioxidants could be a solid alternative to existing therapeutics and a formalized clinical trial could provide insight and benefit.

## 4. Conclusions

In summary, we report a patient who developed thrombocytopenia during treatment with Nintedanib. By reporting this case, we want to raise awareness among clinicians who treat these patients, and pharmacovigilance teams about this rare, reversible, and potentially severe side effect. As Nintedanib becomes more available, and more patients with IPF receive treatment, we can expect more events of thrombocytopenia. The exact mechanism of thrombocytopenia remains unknown, but it could be related to PDGFR inhibition or alternatively immune-mediated effects, and more research is needed to further elucidate the pathophysiology behind this side effect. Thrombocytopenia due to Nintedanib seems to be dose independent and can occur, as illustrated in our case, after 3 months of therapy. We suggest routine platelet monitoring in patients on Nintedanib. 

## Data Availability

All information is publicly available and data regarding this particular patient can be obtained upon request from corresponding senior author.
